# Adapting a breast cancer early presentation intervention for Black women: A focus group study with women of Black African and Black Caribbean descent in the United Kingdom

**DOI:** 10.1111/ecc.13652

**Published:** 2022-07-15

**Authors:** Afrodita Marcu, Lorraine Marke, Jo Armes, Katriina L. Whitaker, Emma Ream

**Affiliations:** ^1^ School of Health Sciences University of Surrey Guildford UK; ^2^ Patient and Public Involvement representative

**Keywords:** Black African, Black Caribbean, breast cancer, early presentation, intervention

## Abstract

**Objective:**

Black women in the United Kingdom are more likely than White women to be diagnosed with advanced breast cancer and have lower survival rates. We consulted women of Black Caribbean and Black African descent in the United Kingdom on how the Promoting Early Presentation (PEP) booklet and intervention could be adapted for Black women to promote early presentation with breast cancer symptoms.

**Methods:**

Focus groups with 22 women of Black African and Black Caribbean descent, of whom five had been treated for breast cancer. The participants were recruited from a large UK breast cancer charity and community settings. Data were analysed using Framework Analysis.

**Results:**

Four themes summarised the participants' views on how the booklet and intervention could be adapted: *Justify the focus on Black women*, *Black people do not talk about cancer*, *Make interventions inclusive and engaging*, and *Engage Black communities to deliver interventions*.

**Conclusion:**

Breast cancer behaviour change interventions need to be more inclusive, illustrate how breast cancer symptoms manifest on black skin, and emphasise that breast cancer is curable to increase awareness and reduce cancer fear. Researchers should involve Black communities in the design and delivery of interventions to address appropriately cultural barriers to early presentation.

## 1 INTRODUCTION

Ethnic inequalities in breast cancer outcomes have been documented in many countries with multicultural populations (Brennan, 2017; Gathani, Chaudhry, et al., 2021; Schwartz et al., 2021), with Black women being particularly at higher risk of mortality. In the United Kingdom, despite breast cancer being less common in Black than White women, Black women (i.e., women of African descent including those women born on the African continent, in the Caribbean, and in the United Kingdom) are more likely to be diagnosed with advanced or metastatic breast cancer and die from the disease than their White counterparts (Jack et al., 2009; Jack et al., 2014; Januszewski et al., 2014). While some genetic factors might account for these differences, such as greater risk of breast cancers with less favourable characteristics (Gathani, Reeves, et al., 2021), higher risk of developing the more aggressive triple negative form of breast cancer (Copson et al., 2014; Jack et al., 2009) and higher likelihood of developing breast cancer at a young age (Copson et al., 2014; Jack et al., 2012), a range of patient factors are also likely to contribute to the poorer outcomes for breast cancer among Black women in the United Kingdom. Understanding how these patient factors might be addressed with behavioural change interventions (e.g., promoting timely help‐seeking) is arguably a first step in addressing breast cancer inequalities.

First, women of Black African and Black Caribbean descent in the United Kingdom have lower breast cancer awareness than White women (Forbes et al., 2011), and lower knowledge of risk factors for breast cancer (Jones et al., 2014; Scanlon & Wood, 2005). Furthermore, some Black women consider breast cancer a “White women's disease,” and some Black women do not feel that messages promoting breast health or breast screening apply to them (Bamidele et al., 2017; Brown et al., 2017; Jones et al., 2015). Second, Black women in the United Kingdom are less likely to report practising breast cancer awareness compared to White women (Forbes et al., 2011; Scanlon & Wood, 2005). They also report lower uptake of mammographic screening (Bamidele et al., 2017; Jack et al., 2009, 2014; Renshaw et al., 2010; Scanlon & Wood, 2005), with main barriers being anxiety over a potential cancer diagnosis, lack of time to attend, and uncertainty over the screening procedure (Bamidele et al., 2017). Third, emotional factors can act as barriers to early presentation with breast cancer symptoms, such as fear of breast cancer (Brown et al., 2017) and fatalistic views about it (Vrinten et al., 2016). A systematic review of barriers to early presentation with breast cancer symptoms among Black women in the United Kingdom found that emotional factors can contribute to delayed presentation: fear of detecting a breast abnormality, fear of cancer treatments, fear of partner abandonment, and cultural factors such as taboos around cancer and perceptions of stigma (Jones et al., 2014).

Few interventions aimed at women of Black African and Black Caribbean descent in the United Kingdom have been developed to date, although some show early promise at increasing awareness of breast cancer and consultations for breast changes (Greenhough et al., 2016). Given the patient factors associated with poorer breast cancer outcomes in Black women, interventions should promote knowledge of symptoms and skills to detect breast changes, and address emotional barriers to symptomatic presentation (Forbes et al., 2011). One breast cancer awareness intervention, *Promoting Early Presentation* (PEP for short) (Burgess et al., 2008) has the potential for behaviour change as it was developed to address the factors associated with delayed presentation with breast changes, such as low knowledge of symptoms and infrequent self‐checking of breasts (Burgess et al., 2008). The PEP intervention is a brief one‐to‐one script‐based communication, accompanied by a booklet with breast cancer awareness messages, delivered by health professionals in a motivational style in one‐to‐one communication with the patient. It addresses emotional barriers to help‐seeking by presenting positively the consequences of breast‐checking and help‐seeking behaviours (Forster et al., 2014). The PEP intervention has been found to increase knowledge of breast cancer symptoms, confidence in checking breasts, and frequency of breast checking (Campbell et al., 2016; Kaushal et al., 2019).

While the PEP intervention has been successfully delivered in primary care to older women (regardless of ethnicity) (Campbell et al., 2016), we do not know whether women of Black African and Black Caribbean descent would find it acceptable and how it would be best delivered to them. Therefore, we consulted women of Black African and Black Caribbean descent on: whether the PEP booklet was suitable, how it could bring about behaviour change, and how its effects could be optimised through adapting it to the target population and context.

## 2 METHODS

### 2.1 Design and materials

Four focus groups ranging from two to eight participants were conducted with women of Black Caribbean and Black African descent to elicit views on how the PEP booklet promoting early presentation for breast cancer could be adapted for and delivered to Black women. The booklet (*Looking after your breasts*) includes: breast cancer symptoms detailed in text and graphics; guidance on how to check breasts for changes; and positively‐framed advice to see the GP promptly if unusual breast changes appear (Forster et al., 2014). The study received a favourable ethical opinion from the University of Surrey Ethics Committee on 10 July 2019 (UEC 2019 058 FHMS).

### 2.2 Recruitment strategy

We aimed to recruit equal numbers of participants with and without personal experience of breast cancer so as to get a broad range of perspectives on how symptomatic presentation with breast changes should be encouraged. Participants were recruited through purposive sampling and snowballing in London, England, between May and October 2019. Breast cancer survivors were sought with assistance from the charity Breast Cancer Now^1^ which, on behalf of the research team, posted the study advert on the charity's patient forum, Breast Cancer Voices.^2^ Participants without a history of breast cancer were approached at the housing association Peabody Trust^3^ through contacts among the research team. Some participants recruited from Breast Cancer Voices mentioned the study to their friends from the group Global Women of Today,^4^ who then volunteered to take part in the research. Women were ineligible if of non‐ Black African or Black Caribbean descent, or if unable to speak English. One participant, a breast cancer survivor, agreed to provide a patient and public involvement (PPI) perspective in the ensuing data analysis and writing up of the findings.

### 2.3 Procedure

The focus groups took place in community settings in London (e.g., public library, church). Participants received a participant information sheet prior to the focus groups informing them that the focus group discussion was audio‐recorded and their contributions anonymised in the transcription. The focus groups lasted on average 72 min (range: 58 to 90 min) and were facilitated by two of the research team. All focus groups started with a general discussion about how women of Black descent in the United Kingdom should be encouraged to present early with signs of breast cancer, and then participants were asked to make suggestions on how the PEP booklet could be adapted and delivered to Black women. Participants were compensated with vouchers for their travel and time taken to participate in the focus groups.

### 2.4 Analytic approach

Data were transcribed verbatim, anonymised, and analysed inductively and thematically using Framework Analysis (Ritchie & Spencer, 2002), whereby we followed a five‐step process: (1) Familiarisation with the data, (2) identifying a thematic framework, (3) indexing, (4) charting, and (5) mapping and interpretation (Gale et al., 2013; Ritchie & Spencer, 2002). The first author read the transcripts and then developed a coding strategy and a thematic framework, which was applied systematically to the data during the indexing stage. In the final stage, the first author generated a more abstract view of the data as themes were more theoretically conceptualised. Field notes helped contextualise the interpretation of the data. The second author, the PPI representative, checked and approved the interpretation of the data. In the quotes below, we have replaced participants' names with pseudonyms and indicate the participants' age, descent, and whether they had a personal history of breast cancer (“survivors”) or not (“no history”).

## 3 RESULTS

### 3.1 Sample characteristics

A total of 22 women participated in four focus groups, age range 39–77, mean = 57. Five breast cancer survivors were recruited from the “Breast Cancer Voices” panel at the charity Breast Cancer Now, while 17 participants with no personal experience of breast cancer were recruited among employees at the Peabody Trust and the women's group Global Women of Today. Thirteen participants were Black Caribbean, six Black African, and three of mixed descent (Asian and Black Caribbean, White and Black Caribbean, White and Black African). Of the 17 participants who did not have personal experience of breast cancer, one had had a previous diagnosis of throat cancer, and more than half (59%, *n* = 10) had a family member or friend diagnosed with breast cancer in the past. Of the 22 participants, 77.3% (*n* = 17) were over the age of 50 and thus eligible for breast cancer screening and of these 100% had participated in screening. The remaining 22.7% (*n* = 5) were not eligible as under the age of 50, however one of these (20%) had participated in screening because she was a breast cancer survivor. Most participants (55%, *n* = 12) were educated to degree or higher degree level, six participants (27.2%) were educated to O Levels/GCSE/higher education below degree level, and two participants (9.1%) had no formal qualifications. Almost half were in full‐time employment (46%, *n* = 10), while a high proportion were retired (32%, *n* = 7).

### 3.2 Constructed themes

Four key themes were developed that captured: participants' views on whether Black women should receive targeted interventions, concerns that stigma can make raising cancer awareness difficult in Black communities, suggestions on how the PEP booklet could be adapted for Black women, and reflections that community engagement is necessary to design effective breast cancer interventions for Black women. In Table 1, we provide additional quotes per theme.

**TABLE 1:** Additional quotes for each theme

Theme	Quote
*Justify the focus on Black women*	A woman is a woman so why it has to be Black cancer and White cancer? […] as long as you have breasts, don't matter if you are pink polka dot stripe. Its breasts. (Cindy, 73, Black Caribbean, no history)
	I felt alone, isolated, because there was no help. And I had a good breast cancer nurse who said “Go to a support group,” so I think that's where my journey started as “This support group, I want to see Black people.” I was the only Black person in the support group. So I challenged Macmillan. I said, “look at your posters, when we pick up leaflets all I see is White. […] you walk into their room, they're all full of White people, and that's what's getting me. Where are the Black nurses? Where are the Black professionals?” (Diane, 57, Black African, survivor)
	I've not had this Black/White issue. I see it more as cancer can happen to anybody, it doesn't matter what colour. […] I'm sure it's just as difficult for a White person to open up about their cancer as for a Black person to open up, because I was born here so I'm more anglicised. (Julie, 55, Black African, survivor)
*Black people do not talk about cancer*	I had breast cancer, nine years ago, 2010, and my first reaction to breast cancer was “Oh, I thought it was a White people's disease.” (Diane, 57, Black African, survivor)
	They don't mention the C‐word. When I had my diagnosis, I only knew of a cousin who had had it before, but then I had genetic testing to see whether it was in the family and I found there were several people in my family that had cancer. […] I did explain to them and said, “Look, it can run in families and I need to know. I have got daughters. […] We need to break this tradition, this culture of not talking about things,” some of them were receptive to it, but others … When it is within your … your value system, it is so rooted in that kind of thing where you cover everything, you don't want to talk about it, then some of them were not receptive. (Lucille, 56, Black Caribbean, survivor)
*Make interventions inclusive and engaging*	I think people need to know that you can survive breast cancer and I think that is one of the huge barriers that this whole thing … The taboo, that people only find out that someone's had breast cancer when they die, that needs to be changed, so there's a greater representation of ordinary Black women being seen as survivors, in order to give people confidence. (Leah, 57, White & Black African, survivor)
	Since my diagnosis, the number of women around my age who have been diagnosed has rocketed, and there's just no education, there's just no awareness. Seriously. I was 27, but I had a friend, she was 29, another one was 32. Its seriously young … I know someone who's 24. (Nadine, 39, Black Caribbean, survivor)
*Engage Black communities to deliver interventions*	I would say maybe if you are trying to get into the awareness of Black women then you have to look at your make‐up offering community engagement because if they don't know about that they are not going to come to you. Maybe you need to look at groups that are out there like women's groups. (Zoe, 61, Black African, no history)
	I think for Black women rising, that [campaign is good] because I think particularly with women of any background, where we rarely put ourselves and our concerns and needs first, and I think Black women even more so. (Ava, 39, Black Caribbean and south Asian, no history)
	And sometimes it's not about just going to the women who are doing work in terms of breast cancer. It's about going to community groups or organisations that are doing particular work. It could be around mental health, but you could have a health day within that and breast awareness could be part of that. (Leah, 57, White and Black African, survivor)
	But there also needs to be a campaign. I'm sure most Black men know that … I would imagine that their awareness that one in four Black men will get prostate cancer is probably a lot greater than how many Black women will know that they'll get breast cancer because you're seeing it all the time, even the TV ad, it's got a Black man on it. (Leah, 57, White and Black African, survivor)
	Mums breast cancer has made me more breast aware. Having said that, my sister—who is younger—I don't think she is that breast aware. It is not something we think about. I don't see anything that represents me in all the literature. Nothing represents me in there [cancer literature]. (Mary, 39, Black African, no history)

#### 3.2.1 Justify the focus on Black women

At the start of three of the four focus groups some participants challenged the focus on Black women, not least because the facilitators were White. Some argued that all women are at risk of breast cancer and therefore awareness‐raising campaigns should target women irrespective of ethnic descent:

Is there a separate protocol for Black women to White women? Are you asking us as individuals here or talking about Black women as a whole? I can only speak for myself. (Alice, 52, Black Caribbean, no history)

However, some participants argued that the focus on Black women was justified because of what they perceived as ethnic bias in healthcare:

We are all women, but the way that we experience the world – particularly the health system, if we look at it in a historical and social context – is markedly different for White women or women from other ethnic backgrounds so I think it is really good that you are doing this. Black women have the highest mortality rates in the country during childbirth and a lot of that is linked to the structural racism that exists in society, but also in the healthcare profession. A lot of the systems are not designed for Black women in terms of facilitating access and engagement. (Ava, 39, Black Caribbean & South Asian, no history)

The breast cancer survivors argued that Black women warrant specific attention in relation to breast cancer as the under‐representation of Black people in cancer materials, cancer patient groups and among healthcare professionals had made them feel isolated during treatment. The participants without a history of cancer agreed that health promotion campaigns generally lack representations of Black women. But while some participants welcomed ethnically inclusive campaigns, others expressed concern that targeted interventions might lead to the stigmatisation of Black communities:

I do not think it has to be targeted at me because I am Black, it has to be targeted at everybody. Make it inclusive […] because you do not want to go through the rigmarole of it's all just happening to Black people. Then it is like a stigma. We are stigmatised again, “only Black people have this.” (Zoe, 61, Black African, no history)

#### 3.2.2 Black people do not talk about cancer

Many participants remarked that in their community “nobody talks about cancer” or mentions the “C‐word”, and that it is common for people to hide their cancer diagnosis even from their closest friends or family:

Some people in the Black community, when it comes to the big C or kidneys or anything else, they do not want anybody to know what is wrong with them. They keep it to themselves. (Patricia, 67, Black Caribbean, no history)

Participants reflected that cultural taboos can make it harder to recognise cancer symptoms, and even lead to the belief that breast cancer does not happen to Black women. The cultural values attached to women's breasts as a symbol of femininity and fertility can dissuade women from practising breast awareness:

It is this thing about, from my culture, which is Black African, anything that has to do with you not having any of the glands that define you as a woman is almost taboo. You might have a lump and your breast might get taken off … I do not even want to check. I do not want to know. (Iris, 44, Black African, no history)

One breast cancer survivor had asked her wider family to disclose cancer diagnoses in the family so that she could know about genetic risks and protect her own two daughters. Another breast cancer survivor had experience as a campaigner to raise cancer awareness among Black communities and had noticed that people of Black Caribbean or Black African descent are reluctant to engage with cancer topics:

Although the purpose was to try and make it more inclusive, in terms of people from an African and Caribbean background, it was still a struggle to get enough people of colour on the course. […] When I've been out on the road, I get people … “I do not want to know about that, if I'm going to die I do not want to know about it.” (Leah, 57, White & Black African, survivor)

#### 3.2.3 Make interventions inclusive and engaging

While the first theme captured the participants' debate as to whether women of Black Caribbean and Black African descent warrant special attention in relation to breast cancer, the third theme captured their suggestions on how the PEP intervention and interventions in general could be developed to target effectively women of Black Caribbean and Black African descent. The participants suggested changes to the PEP booklet regarding content (e.g., statistics on breast cancer among Black women), inclusivity, and culturally relevant representation (e.g., black breasts, typical Black names), and changes to appearance and layout. Some participants suggested that the slogan *Early detection means best protection* should be on the first page to increase appeal and positive tone, while others suggested that case studies and statistics on cancer among Black women would increase engagement:

Having statistics and a bit more about Black culture … Because if it is not there, that's how people detach themselves from [leaflets]. (Irene, 58, White and Black Caribbean, no history)

The breast cancer survivors argued that the booklet should emphasise that breast cancer is curable and should represent Black women as survivors of breast cancer, and suggested a more engaging title as *I survived breast cancer because I looked after my breasts*. It was thought that the booklet should mention the possibility of cancer occurring in young women before they are eligible for breast cancer screening. While some participants did not see the need for cultural adaptation because “*a breast is a breast*”, others thought it should include representations of how breast cancer symptoms manifest on black skin:

There is something that came to mind when I was looking at this from page 3, when it said redness of your breast skin. I was thinking, depending on the colour of skin it might not show redness. How would that work if I am really dark? What would I know when it says redness of your breast skin? (Zoe, 61, Black African, no history)

Furthermore, it was suggested that the booklet should include names typical of Black women, so that it would resonate with the target audience:

Going back to even things like the pictures, but everybody on page 6 … There is a “Barbara,” a “Pat,” a “Mary.” [laughter] In my head I am thinking they are all White women. Maybe if we had “Zeinab” … It is silly little things like that, of culture, that you then relate to yourself. (Iris, 44, Black African, no history)

#### 3.2.4 Engage Black communities to deliver interventions

Regarding how the adapted PEP booklet could be delivered to Black women, the participants stressed the importance of sustainable community engagement as a first step in raising awareness of breast cancer as it would increase trust in the message and uptake of precautionary measures. Some participants also suggested building on existing cancer awareness campaigns aimed at Black women, such as the ‘Black Women Rising’ project led by the breast cancer survivor Leanne Pero:

It's about connecting with activists and people who are doing work on the ground and people that can really connect with certain communities, and it's about building relationships with those people because they are going to bring people. […] The women trust them, particularly if it's in certain communities. So it is about networking, building relationships locally with key people in the community. (Nadine, 39, Black Caribbean, survivor)

The suggestions for community engagement went beyond the mere distribution of the PEP booklet or the delivery of the PEP intervention and indicated a need to engage more deeply with Black communities to deliver cancer awareness and education. Some participants who were breast cancer survivors suggested that such campaigns would benefit from people with direct experience of cancer:

You probably need people like me who have gone through it that they can identify with and that they can think it is okay to talk about it. They need that identifying person so that it is relevant. (Lucille, 56, Black Caribbean, survivor)

The participants also suggested social media, for example, Twitter, as channels for raising breast cancer awareness, especially among younger people. Some participants were against the delivery of the intervention in primary care venues because these are usually associated with delivering bad news. Other participants suggested modelling the breast cancer intervention on prostate cancer awareness campaigns targeting Black men, which were familiar to many participants.

## 4 DISCUSSION

### 4.1 Main findings

In this study we consulted UK women of Black African and Black Caribbean descent on how a current breast cancer intervention, *Promoting Early Presentation* (PEP) (Burgess et al., 2008) could be adapted and delivered to women of Black African and Black Caribbean descent in the United Kingdom. We will summarise the results and discuss their implications.

The first theme, *Justify the focus on Black women*, shows that there needs to be appropriate communication and justification for why Black women warrant special attention in relation to breast cancer. While some participants welcomed the positive discrimination as a recognition of health inequalities, others resisted being categorised as “at risk” on the basis of ethnicity, similarly to participants in other studies (Brown et al., 2019). The findings suggest that there may be an inherent paradox in how Black women may wish to receive breast cancer advice, as there is arguably a fine balance to strike between designing culturally sensitive health promotion materials while at the same time avoiding “othering” or stigmatising certain communities.

The second theme, *Black people do not talk about cancer*, summarised the participants' experience of not talking, or being talked to, about cancer in their families and wider community. The taboo and stigma associated with cancer, as well as the perception that cancer only occurs to White people, led to concerns that it would be difficult to engage with Black communities on this topic. Our findings echo previous research on promoting uptake of cancer screening in BAME populations in the United Kingdom (Baird et al., 2021; Bamidele et al., 2017; Thomas et al., 2005). Our findings also echo research on Nigerian and Ghanaian people's perceptions of cancer in the United Kingdom, which revealed secrecy around a cancer diagnosis as a frequent cultural practice among these two ethnic groups in the United Kingdom (Ehiwe et al., 2012).

The suggested changes to the content of the PEP booklet, captured under the third theme, *Make interventions inclusive and engaging*, pertained both to factual information (e.g., breast cancer is curable) and to culturally relevant images and language. The suggestion that more emphasis should be put on Black women as survivors of breast cancer to reduce fear of cancer resonates with past research which found that people of Black African and Black Caribbean descent in the United Kingdom hold more fatalistic beliefs about cancer than White people (Vrinten et al., 2016).

The fourth theme, *Engage Black communities to deliver interventions*, captured the participants' thoughts around the distribution of the adapted PEP booklet and it revealed the need for wider engagement with Black communities about cancer awareness and prevention. Community‐based networks have been recognised before as suitable channels for delivering breast cancer awareness interventions (Bamidele et al., 2017). The findings suggest that it is important to use established routes of community engagement before delivering health promotion materials. For example, a recent cancer awareness programme in the United Kingdom has demonstrated that peer‐led community interventions can be effective at increasing knowledge of cancer screening programmes, recognition of cancer symptoms, and recognition of risk factors for cancer (Williams et al., 2020).

### 4.2 Study strengths and limitations

One strength of this study is that we recruited women of Black African and Black Caribbean descent from a variety of backgrounds, including women with a past history of breast cancer which provided insight into what it means to be diagnosed and treated for breast cancer as a Black woman in the United Kingdom. We also included women who have tried to raise awareness of breast cancer in their respective community, and their experience provided us with insight into potential barriers. A further strength of this study is the inclusion of a participant as a co‐author and PPI representative, who has validated our interpretation of the data and the conclusions we have drawn. Our study thus contributes to reducing the under‐representation of women of Black African and Black Caribbean descent in PPI in cancer research (Pii et al., 2019).

One limitation of this study is that some of the participants who were breast cancer survivors (*n* = 5) were recruited via the charity Breast Cancer Now, which may not be representative of breast cancer charities in the United Kingdom or indeed of Black women's experience of breast cancer. Another study limitation is that we did not recruit participants according to age or to generational status (i.e., first/second/third etc. generation Black African/Caribbean in the United Kingdom). This may have indicated generational and cultural differences in cancer beliefs or behaviour, like uptake of breast cancer screening, which may have influenced views on how the PEP intervention could be adapted and delivered for different age groups within these two communities. A further limitation is that we recruited participants only in London, and thus our sample is not nationally representative. Although one of the interviewees became part of the project team and co‐author, we did not include PPI or involve any women of Black African or Black Caribbean descent in the earlier design and conduct of the focus groups. Had we done so, we may have minimised the sense of being othered when discussing differences between Black and White women regarding breast cancer outcomes. The relatively high educational status of our sample is also a potential limitation and as such our study may not have captured how other barriers to early presentation (e.g., language and transport) could be addressed through interventions. Last but not least, the standard ethnic categories we used in the demographic questionnaire may not have reflected how participants choose to identify themselves; for example, “Black British” could have been included as a category to minimise the sense of feeling “othered” during the research process.

### 4.3 Implications for research and practice

While further research and community input are needed to design culturally sensitive and inclusive interventions, attention also needs to be paid to the mode and timing of intervention delivery. The current results suggest that women of Black Caribbean or Black African descent would prefer peer‐led interventions or interventions delivered in the community. However, future research should also explore the feasibility and acceptability of delivering breast cancer awareness interventions in healthcare settings, which may have the potential of reaching larger numbers of women.

Regarding the inclusion of women of Black Caribbean or Black African descent in breast cancer research, our approach of using snowballing, known contacts, and charities like Breast Cancer Now showed it was feasible to recruit women from these communities, with and without a history of breast cancer. We found it easy to establish trust with the participants once we explained the purpose of our study and the need to address ethnic inequalities in breast cancer. Our participants were keen to share their views and experiences and saw our research as worthwhile. Had recruitment proved to be difficult, we would have reached out to other breast cancer charities and community groups serving the needs of people of Black African and Black Caribbean descent.

### 4.4 Conclusions

The participants in our study viewed the PEP intervention positively and largely agreed that culturally tailored interventions would be acceptable for raising awareness of breast cancer among women of Black Caribbean and Black African descent in the United Kingdom. While the PEP booklet provided a vehicle for discussion of wider issues of cancer awareness, beliefs, and cultural barriers around cancer, the focus groups indicated that the original PEP intervention may not be entirely suitable for our target intervention groups. Further research and community involvement are needed to adapt existing interventions like the PEP in a culturally sensitive manner with extensive input from women of Black Caribbean and Black African descent.

## CONFLICTS OF INTEREST

The authors declare that they have no conflict of interest.

## Data Availability

Research data are not shared.

## ENDNOTES

1 Breast Cancer Now is a UK charity that funds research on breast cancer and offers support to people affected by breast cancer.

2 Breast Cancer Voices is a patient community and forum affiliated with the charity Breast Cancer Now.

3 Peabody Trust is a London housing association that provides housing and a range of community programmes.

4 Global Women of Today is a UK Coffee Morning Group for Women of All Nations who are feeling lonely and Isolated.
